# A Renal Calculus

**Published:** 1879-06

**Authors:** Frederick Peterson

**Affiliations:** Resident Physician, Buffalo General Hospital


*ART. III.—A Renal Calculus.* Service of Dr. C. Diehl. Reported by Frederick Peterson, Resident Physician, Buffalo General Hospital.

Mrs. B., aet. 62, married, German, was admitted into the hospital April 14th, 1879. Patient had suffered about fourteen weeks from dysuria and unendurable smarting in the urethra. Her health during this period had gradually failed, and when she entered the hospital was unable to eat without immediate vomiting, or to sleep without pain. Renal or cystic disorder was suspected. On examination the urine was found to contain albumen in great quantity. Complained of no pain in the region of the kidneys, but only of the urethral irritation. Was given Acid, Tannic, gr. V., Tinct. Opii. gtt. XX., three times daily with Spiritus Frumenti ℥ twice daily.

April 25. Her vomiting has ceased and she is entirely free from pain. The laudanum is discontinued. She eats but little; milk, beef tea and the stimulant being her only nourishment. Acted all day in a very eccentric manner. Not confined to bed but staggers as she walks about.

May 2. Has become insane. She broke a window, drove other patients from the ward, and finally, after further violent demonstrations, she fell exhausted against her bed. It was found necessary to put on a strait-jacket. Refused food and medicine, exclaiming that we desired to poison her.

May 3d. Still unmanageable. Keeps her jaws firmly clenched when any attempt is made to feed her. Finally resorted to elastic catheter introduced into the œsophagus through the nasal passages, and with the assistance of a Davidson syringe, a pint of milk and two gills of beef tea found entrance into the stomach.

May 4th. There is no diminution in her obstinacy in refusing nourishment and stimulants. She received her food in the same effective and unique manner as in the day before.

May 5th. Not demonstrative; struggles in her bed to the extent of destroying two strait-jackets. Towards evening she became quiet, and still later the *facies Hippocratica* made its appearance.

May 6th. Died quietly at 3.40 A. M. from exhaustion.

May 7th. A *post mortem* examination was made, several members of the hospital staff being present. The abdomen was opened. Had great difficulty in removing kidneys, from adhesions. The left kidney was so much atrophied that we experienced great trouble in finding it. It weighed two ounces, was very soft, and presented externally a circular depression one inch in diameter. On dividing it laterally, a quantity of black disintegrated pus exuded, and found entire degeneration of cortical substance, there being four or five small cavities in the organ. The right kidney was much hypertrophied, weighed twelve ounces, and an incision brought to light a condition of the gland similar to that of the opposite side, but the abscesses were larger and contained a greater quantity of purulent matter. The most singular and interesting event of the examination was the discovery of a calculus in the pelvis of the kidney of extraordianry proportion, and perfectly filled to the infundibula and calices. The calculus, irregular and somewhat triangular in form, weighed one hundred and fifty grains. Its greatest dimension was one and three-quarters inches, its width one and five-eighths, and its thickness seven-eighths of an inch. It was composed of the phosphates. The walls of the bladder were thickened, and the sac was full of disorganized urine and pus. Her mania was probably due to the toxological effect of non-elimination of urea. A calculus resembling this is made mention of in the *Medical Record* of May 3d, 1879, but was one found in dissection, where the previous history of the subject was unknown. The writer is inclined to consider this case worthy of record.

